# Stereotactic Body Radiation Therapy for Prostate Cancer Using Tomotherapy With Synchrony Fiducial Tracking

**DOI:** 10.7759/cureus.40778

**Published:** 2023-06-22

**Authors:** Takashi Shintani, Shimpei Anami, Keisuke Sano, Wataru Okada, Masao Tanooka

**Affiliations:** 1 Department of Radiotherapy, Takarazuka City Hospital, Takarazuka, JPN

**Keywords:** intrafraction motion, tomotherapy, sbrt, stereotactic body radiation therapy, prostate cancer

## Abstract

Numerous prospective and retrospective studies have demonstrated the efficacy and safety of stereotactic body radiation therapy (SBRT) for prostate cancer. Since SBRT utilizes a very tight margin, management of intrafraction prostate motion is necessary. As a real-time motion tracking and correction system (Synchrony; Accuray, Sunnyvale, CA) has been introduced in the newer platform of tomotherapy (Radixact; Accuray), Radixact can deliver tracking SBRT. In the case report, we present the first clinical experience with prostate SBRT using tomotherapy with Synchrony fiducial tracking.

## Introduction

Stereotactic body radiation therapy (SBRT) is a high-precision radiation treatment that delivers large, daily doses of radiation in a small number of fractions. Several studies have demonstrated the safety and efficacy of SBRT for prostate cancer [1-4]. Moreover, SBRT is now regarded as a radiation regimen for all-risk clinically localized prostate cancers [5].

Intrafraction prostate motion can be significant owing to a combination of factors such as muscle tension or relaxation, respiratory motion, bowel movement, and rectal and bladder filling [6,7]. As prostate SBRT utilizes a very tight margin, the management of intrafraction motion is considered extremely important. Prostate SBRT can be delivered using various types of platforms, such as robotic-arm-mounted linear accelerators (LINAC), gantry-mounted LINAC, and magnetic resonance imaging (MRI)-guided LINAC. Although SBRT using CyberKnife (Accuray, Sunnyvale, CA) or an MRI-guided LINAC has a theoretical advantage in managing intrafraction motion, only gantry-mounted LINAC is commonly available at treatment centers with limited resources. Therefore, an effective strategy to manage intrafraction motion during prostate SBRT using gantry-mounted LINAC is required.

Radixact (Accuray) is the latest version of the helical tomotherapy delivery platform, and the real-time motion tracking and correction system, Synchrony (Accuray), which was originally designed for CyberKnife, was clinically incorporated into the Radixact system in 2019. Thus, Radixact is capable of delivering tracking SBRT. Herein, we report our first clinical experience of prostate SBRT using Radixact with Synchrony fiducial tracking.

## Case presentation

A 77-year-old man was diagnosed with prostate cancer (cT2aN0M0, prostate-specific antigen concentration 5.61 ng/mL, Gleason score 4 + 5, 4/12 biopsy cores positive, involving the right peripheral zone). The medical history of the patient was notable for heart disease (postmitral valvuloplasty for mitral regurgitation and post-pacemaker implantation for sick sinus syndrome and atrial fibrillation). He routinely consumed the prescribed anticoagulants and antiplatelets. The patient elected definitive treatment with prostate SBRT and long-term androgen deprivation therapy (ADT).

Treatment preparation and planning

The transrectal ultrasound-guided transperineal insertion of a hydrogel spacer between the Denonvilliers’ fascia and anterior rectum was performed using a commercially available system (SpaceOAR System; Boston Scientific, Marlborough, MA) three months after the initiation of ADT. On the same day, three fiducial markers (Acculoc; CIVCO Medical Solutions, Kalona, IA) were placed in the prostate.

Radiation therapy planning computed tomography (CT) and MRI scans were performed three weeks after the placement of the hydrogel spacer and fiducial markers. An empty rectum and a full bladder were required for the simulation and each treatment session.

**Figure 1:**
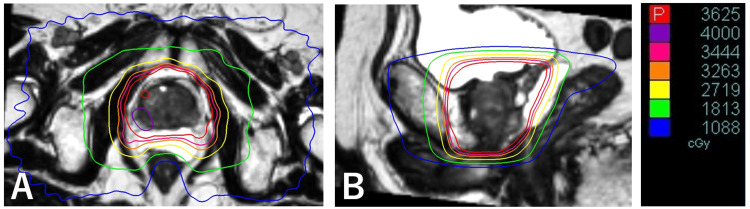
Dose distribution of prostate SBRT The prostate SBRT treatment plan is displayed on fused T2-weighted MRI on the representative (A) axial and (B) sagittal views. Abbreviations: SBRT, stereotactic body radiation therapy; MRI, magnetic resonance imaging

The MRI scan was fused to the CT scan using a fiducial marker match. The clinical target volume (CTV) included the prostate and proximal 1 cm of the seminal vesicles. The CTV to planning target volume (PTV) expansion measured 5 mm isotropically, except for 3 mm posteriorly. The dominant intraprostatic lesion (DIL) was delineated by comparing the planning scans with staging MRI (T2-weighted and diffusion-weighted images) slice-by-slice. A PTV boost was defined as DIL with no margin. The prescribed dose was 36.25 Gy in five fractions to the PTV with a simultaneous integrated boost of 40 Gy in five fractions to the PTV boost. The radiation dose was prescribed so that the prescription isodose line encompassed the PTV. The planning goal of the target coverage is as follows; D95% of PTV ≥ 36.25 Gy, D98% of PTV ≥ 34.4Gy, maximum dose to PTV ≤ 45 Gy, and D95% of PTV boost ≥ 40 Gy, where Dx% of Y means the minimum dose to X% of Y. The beam-on time was 8 min 13 s. The dose distribution in a representative slice is presented in Figure 1.

Treatment delivery

SBRT treatment was delivered on alternate days by Radixact with Synchrony fiducial tracking for non-respiratory irregular motion. Radixact Synchrony is equipped with a pair of kV (X-ray tube voltage) radiography and a flat detector panel mounted on the gantry. The target position was calculated based on the fiducial marker position detected by successive two-dimensional (2D) kV radiographs, and the target motion was compensated by the jaw sweeping in the longitudinal direction and multileaf collimator (MLC) shifting in the lateral and vertical directions.

**Figure 2:**
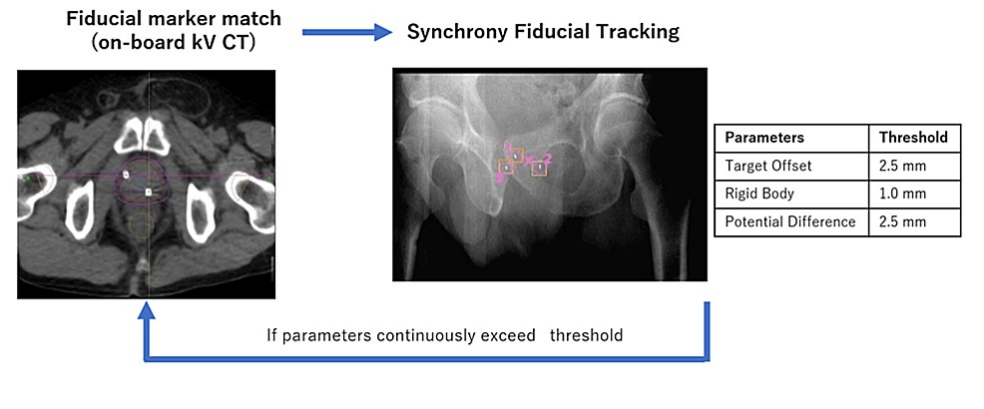
Workflow of treatment delivery On-board kV CT is used for the initial setup to match the three fiducial markers to the positions on the planning CT. The translational correction and the roll rotational correction can be made by the couch shift and by the adjustment of the gantry start angle while the pitch and yaw rotational error cannot be corrected. Intratreatment 2D kV radiographs are obtained six times (25, 75, 155, 205, 255, and 335 degrees) per one gantry rotation. Several parameters to control tracking SBRT delivery are shown, and if these parameters continuously exceed the threshold, we acquire another onboard kV CT to correct the motion shift. Abbreviations: SBRT, stereotactic body radiation therapy; CT, computed tomography; kV, x-ray tube voltage

The workflow of SBRT is presented in Figure 2.

**Figure 3:**
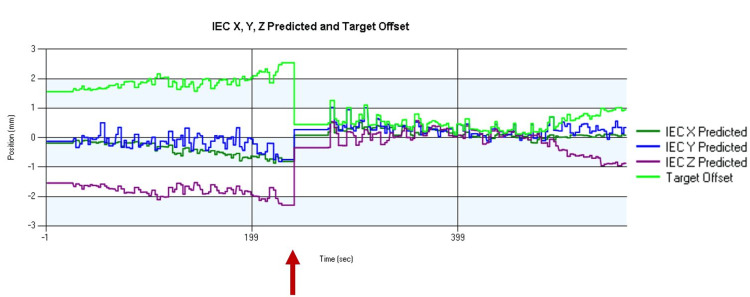
Example of the delivery report of tracking SBRT International electrotechnical commission X (lateral), Y (longitudinal), and Z (vertical) displacement of fiducial markers from the position in the planning CT at fraction four is displayed. The target offset exceeded the threshold level (2.5 mm) due to a gradual shift (red arrow), and we acquired onboard another kV CT to correct the position and resumed the treatment. Abbreviations: SBRT, stereotactic body radiation therapy; CT, computed tomography; kV, x-ray tube voltage

The initial patient setup was performed through fiducial matching using helical kV CT. Intrafraction 2D kV radiographic images were acquired six times per gantry rotation to monitor the locations of the fiducial markers. The thresholds of the parameters to control the tracking SBRT delivery were as follows: 2.5 mm for potential difference (three-dimensional (3D) distance error when the model is used), 2.5 mm for target offset (predicted target position change from the position in the planning CT), and 1 mm for a rigid body (maximum fiducial pair distance difference from the planning CT), respectively. If the parameters exceeded the thresholds, the radiation delivery was paused, and the intrafraction kV CT was acquired to correct the target shift. A representative treatment report is explained in Figure 3. The number of intrafractional kV CT acquisitions to correct the target shift was zero to one in each treatment session.

SBRT treatment was accomplished without any difficulties, and the patient tolerated the treatment well.

## Discussion

The management of intrafraction prostate motion is important for the precise delivery of prostate SBRT. Intrafraction motion can be broadly divided into gradual shifts and abrupt transient excursions, and the resulting motions occur mostly in the vertical and longitudinal directions. Prolonged treatment time has been correlated with a high frequency of significant intrafraction motion [8]. Therefore, intrafraction motion management should be strongly considered when delivering prostate SBRT using tomotherapy because the delivery of the treatment takes approximately 10 minutes.

With the introduction of the Synchrony system, tomotherapy can be used to track SBRT [9,10]. By using the system, the 3D fiducial position is calculated based on successive 2D kV radiographs, and the translational motion can be compensated by jaw sweeping in the longitudinal direction and MLC shifting in the lateral and vertical directions. Thus, there is a limitation in motion compensation in the lateral or vertical directions, as any motion less than 3.125 mm (half the width of the MLC width) cannot be compensated. The PTV posterior margin was only 3 mm, and we considered that it would be better to pause treatment before the motion amplitude reached the PTV minimum margin level to avoid underdosing the posterior part of the CTV. In other words, our strategy is to use the Synchrony system primarily to confirm that the target is well within the PTV rather than for tracking large motions.

The study had some limitations. One may argue that our strategy leads to frequent treatment pauses, intrafraction kV CT for realignment, and longer treatment time if the prostate motion shows frequent large excursions. However, from the viewpoint of delivery dose accuracy, some phantom studies have revealed that it is better to pause and resume treatment after couch correction in cases of rapid large excursions than to continue treatment [11]. In addition, we believe that the occurrence of such motions, primarily caused by gas movement, can be avoided using the empty rectum protocol.

## Conclusions

The beam-on time of prostate SBRT using tomotherapy is approximately 10 minutes, and the long treatment time also requires the management of prostate motion. The Synchrony system, incorporated into Radixact, enables intrafraction monitoring and tracking. In this report, we describe the successful case of prostate SBRT using Radixact with Synchrony. Further studies are needed to assess the applicability and clinical outcomes of our approach in larger cohorts.
